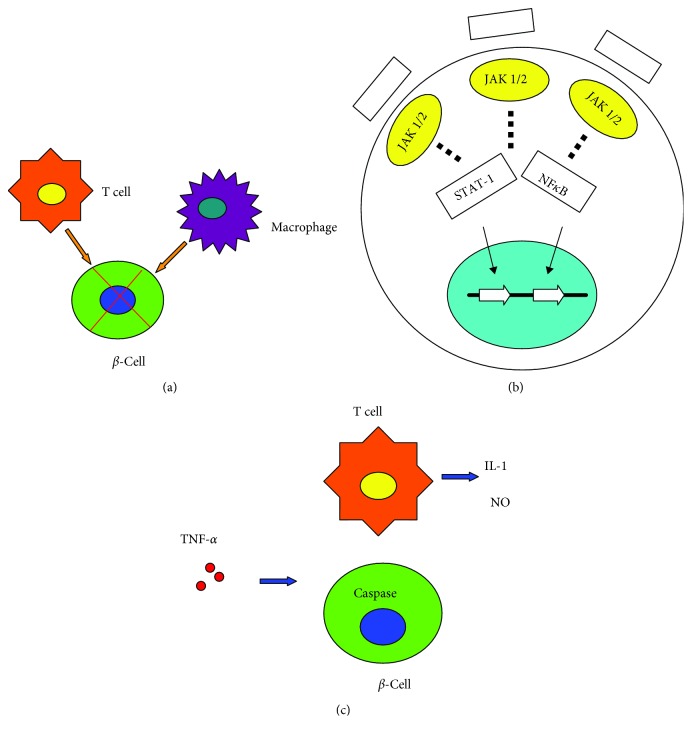# The Association between Depression and Type 1 Diabetes Mellitus: Inflammatory Cytokines as Ferrymen in between?

**DOI:** 10.1155/2019/2987901

**Published:** 2019-03-28

**Authors:** Kexin Wang, Fangna Li, Yixin Cui, Chunhui Cui, Zhenzhen Cao, Kai Xu, Shuhong Han, Ping Zhu, Yu Sun

**Affiliations:** ^1^Department of General Surgery, Qilu Hospital of Shandong University, Jinan, Shandong 250012, China; ^2^Cheeloo College of Medicine, Shandong University, Jinan, Shandong 250012, China; ^3^Department of Endocrinology, Qilu Hospital of Shandong University, Jinan, Shandong 250012, China; ^4^Department of Endocrinology, The First People's Hospital of Pingyuan County, Pingyuan, Shandong 253100, China; ^5^Department of Medicine, Division of Rheumatology & Clinical Immunology, University of Florida, Gainesville, FL 32610-0275, USA; ^6^Department of Ophthalmology, College of Medicine, University of Florida, Gainesville, FL 32610-0284, USA

## Abstract

The depression incidence is much higher in patients with diabetes mellitus (DM), and the majority of these cases remain under-diagnosed. Type 1 diabetes mellitus (T1D) is now widely thought to be an organ-specific autoimmune disease. As a chronic autoimmune condition, T1D is characterized by T cell-mediated selective loss of insulin-producing *β-*cells. The age of onset of T1D is earlier than T2D, and T1D patients have an increased vulnerability to depression due to its diagnosis and treatment burden occurring in a period when the individuals are young. The literature has suggested that inflammatory cytokines play a wide role in both diseases. In this review, the mechanisms behind the initiation and propagation of the autoimmune response in T1D and depression are analyzed, and the contribution of cytokines to both conditions is discussed. This review outlines the immunological mechanism of T1D and depression, with a particular emphasis on the role of tumor necrosis factor-*α* (TNF-*α*), IL-1*β*, and interferon-*γ* (IFN-*γ*) cytokines and their signaling pathways. The purpose of this review is to highlight the possible pathways of the cytokines shared by these two diseases via deciphering their cytokine cascades. They may provide a basic groundwork for future study of the possible mechanism that links these two diseases and to develop new compounds that target the same pathway but can conquer two diseases.

## 1. Depression and Diabetes Mellitus

Studies have implicated an increased prevalence of depressive disorders comorbid with anxiety in patients with diabetes mellitus [1]. Depression has an adverse effect in DM and not only significantly increases the risk for other complications but also a predictor for an earlier incidence of complications [2].

Although the cooccurrence of depression in DM could be attributed to a variety of factors, such as the psychological impact of the disease, a potential common genetic susceptibility and cerebrovascular insufficiency that results from neuroimmunological and neuroendocrinical pathways may be present, as well as microvascular brain lesions due to DM [3]. However, the underlying mechanisms concerning the high cooccurrence between these two diseases are not fully profiled yet. Inflammatory cytokines have been found to contribute to the development of depression in both medically healthy and medically ill individuals, including DM [4, 5]. Therefore, we outlined the involved cytokines in both diseases and their possible regulation pathways individually in this review and provided a general idea as to whether circulating cytokines bridge the pathogenesis of these two diseases. Through this summary, we may map out a pathway for the future studies of these two diseases using some common pathways shared by them for disease initiation and progression. We also evaluated the evidence that associates rheumatoid arthritis with depression, assessing the immune and molecular responses to inflammation.

## 2. Depression, Inflammation, and Cytokines

### 2.1. Systemic Inflammation and CNS Depression

Depression is one of the leading causes of disability worldwide, and its incidence is expected to increase. According to the WHO, depression will be the leading cause of disability globally around the year 2030. Although extensive research has been invested into the mechanisms of this disease, the rate of depression is still rising, especially in industrialized countries. The cooccurrence of immune-mediated inflammatory diseases with depression has been well acknowledged in the literature [6]. Persistent low-grade inflammation in peripheral tissues is one of the known predisposing factors in major depression. The overlap timeframes of peripheral and CNS inflammatory responses under immunological conditions raise the possibility of shared pathophysiological mechanisms [6]. Chronic inflammation exacerbates the sickness and depression-like behaviors in patients, which indicates the possible negative effects of inflammatory cytokines on monoaminergic neurotransmission, neurotrophic factors, and measures of synaptic plasticity [7]. Meanwhile, emerging evidence has suggested a strong association between depression and inflammatory processes, and clinical studies have implicated antidepressant treatment effects being boosted with anti-inflammatory agents, both as add-on treatment and as monotherapy [8]. All of the evidence so far has suggested that depression may facilitate inflammatory reactions and cytokine alterations and that inflammation could promote the development of depression. The possible intertwining interactions between them may form a bidirectional loop. Changes in cytokines could be one of the key factors that predispose one to behavioral changes by affecting neurotransmitters, ion channels, and receptors. Therefore, peripheral inflammation may be a key component that closely collaborates with neurotransmitter dysregulation in CNS and adversely affects brain function, leading to psychiatric disorders, including depression.

### 2.2. Cytokines and CNS Depression

The previous well-established abnormality theory of depression in monoamine systems has been challenged so far, and some studies of functional neuroimaging revealed evidence supporting the relationship between structural/functional anomalies in the brain and a parallel increase of circulating inflammation markers [9]. A handful of previous studies have shown that increases in circulating inflammatory cytokines, including IL-1, TNF-*α*, and IFN-*γ*, have been validated in depression [4]. Subjects with depression also demonstrated exacerbated inflammatory responses to stressful impact via upregulation of cytokine levels in the CSF [10], which suggests that circulating cytokines and depression affect each other in a bidirectional way. The changes of those above cytokines in T1D may be the potential culprits that are responsible for the development of depression in DM patients.

### 2.3. TNF-*α*

As one of key players of innate immunity, TNF-*α* is also a physiological regulator of homeostatic cell proliferation, differentiation, and programmed cell death in the CNS [11]. There is emerging evidence implying that TNF-*α*-producing macrophages play a key role not only in the neurodevelopment but also in the pathophysiology of various neuropsychiatric conditions, including depression [12]. In a recent clinical study, the role of TNF-*α* in the development of depression and anxiety in a systemic inflammatory disease, systemic lupus erythematosus (SLE), was explored. The results demonstrated that sera TNF-*α* levels are increased in SLE patients with mood and anxiety disorders. In SLE, TNF-*α* levels in the sera of SLE patients were independently associated with mood disorders that developed in the patients [13]. These findings suggest the presence of TNF-*α* and other cytokines may be the immunological basis for depression in SLE. Preclinical study has shown that the deletion of TNF-*α* is associated with antidepressant-like effects in behavioral tests in mice in comparison with wild-type mice [14], which further implicates TNF-*α* as having an important role in the development of depression.

### 2.4. IFN-*γ*

As a crucial player in innate and adaptive immune responses for many autoimmune diseases, including TID, IFN-*γ* has recently been recognized to play an important role in stressor-related psychological pathology [15]. Along with other cytokines, IFN-*γ* levels in the serum of depressed patients were found to be significantly increased compared to their counterparts in a control group in a previous clinical study [16]. In line with the above study, an elevated plasma IFN-*γ* level was observed in another clinical study with adolescent subjects with depression [17]. More importantly, in the latter study, the researchers further demonstrated the IFN-*γ* level could be regulated to a normal level after patients underwent effective antidepressant treatment and their symptoms were under control [18]. These results suggest that changes of IFN-*γ* level are closely associated with depression, and regulation of cytokine expression in patients should be one of the therapeutic targets of treatment. Some preclinical studies also had consistent findings that supported the hypothesis that IFN-*γ* is a crucial mediator in the pathogenesis of depression [19]. Mice infected with an IFN-*γ* adenovector demonstrated anhedonic-like symptoms [20]. Meanwhile, several studies showed that some commonly used antidepressants that attenuate depressive symptoms antagonize IFN-*γ* signaling [21, 22].

### 2.5. IL-1*β*

Recent reports have verified that there are increases in proinflammatory cytokine IL-1*β* in both younger and elderly adults with major depression [23]. Moreover, these studies also provided evidence that the serum IL-1*β* level of patients is strongly correlated with the severity of their depression and the duration of the current depressive episode. In a preclinical study, increases of IL-1*β* in serum were associated with acute stress [23]. In addition, the IL-1*β* level in the CSF of acutely depressed and unmedicated patients was found to be higher than in the control group [24]. Meanwhile, some studies indicated that antidepressant treatment significantly decreased the expression of IL-1*β* levels in depression patients. In animal models, IL-1*β* treatment could effectively cause depressive-like behaviors but treatment with IL-1RA resulted in antidepressant-like effects [25]. In neurodegenerative diseases, such as Alzheimer's disease (AD), IL-1*β* is also actively responsible for the development of depressive symptoms [26]. IL-1*β* plasma levels are also significantly associated with cooccurring depressive symptoms in temporal lobe epilepsy [27]. These findings imply that the IL-1*β* signaling pathway may be one of the common pathways shared by depression and other systemic diseases.

## 3. Autoimmune Type 1 Diabetes Mellitus and Cytokines

Diabetes mellitus (DM) is a chronic progressive metabolic disorder characterized by hyperglycemia with disturbances of carbohydrate, fat, and protein metabolism, mainly due to defects in insulin secretion, insulin action, or both [28]. DM is one of the oldest recognized diseases, first reported 3000 years ago by the ancient Egyptians [29]. Diabetes is a global public health problem and is now emerging as a pandemic [30]. Diabetes-related complications include cardiovascular disease, nephropathy, neuropathy, retinopathy, and lower-extremity amputation [31, 32]. It results in a significant increase in morbidity and mortality, placing heavy economic burdens on families and health care systems. Both the number of cases and the prevalence of diabetes have been steadily increasing over the past few decades. The World Health Organization (WHO) has estimated that in 2013, approximately 346 million adults were living with diabetes worldwide, compared to 108 million in 1980, and this number is predicted to have almost doubled by 2030 [33].

There are two major forms of diabetes mellitus: type 1 and type 2 diabetes (T1D and T2D). T1D, also known as juvenile or insulin-dependent diabetes mellitus (IDDM), is typically caused by an absolute deficiency of insulin secretion. T2D, previously known as adult or noninsulin-dependent diabetes mellitus (NIDDM), is the result of a progressive deficiency of insulin secretion on the background of resistance to the action of insulin in both peripheral tissues (e.g., muscle and adipose tissues) and *β-*cells [34].

T1D accounts for 5–10% of the total cases of diabetes worldwide [34]. The disease can occur at any age but is often observed in adolescence and early adulthood. T1D is a chronic autoimmune condition that is characterized by T cell-mediated selective loss of insulin-producing *β*-cells in the islets of Langerhans of the pancreas. Both genetic and environmental factors contribute to precipitating the disease, and the outcomes of the pathological process depend on multiple interrelated factors. The majority of cases (70–90%) are type 1A diabetes, which is attributable to an autoimmune-mediated destruction of beta cells, while type 1B diabetes (idiopathic) represents a small minority of cases whose specific pathogenesis remains unclear [35]. Although T2D has become increasingly prevalent in children and adolescents, T1D continues to be the most common type of diabetes in this population group, [36]. The understanding of the disease has evolved over the past decade or so, and the age at symptomatic onset is no longer the limiting factor [35]. Children and adolescents with T1D typically present with a hallmark triad of symptoms (i.e., polydipsia, polyphagia, and polyuria), along with overt hyperglycemia, but to a lesser degree in adults [37]. Patients with T1D require lifelong insulin treatment and are prone to ketoacidosis. Technological innovations in insulin pumps and continuous glucose monitors have positively impacted the quality of life of T1D patients since they require lifelong insulin administration. However, effective prevention or cures for T1D remain elusive. Globally, the quality of diabetes management remains uneven. Therefore, it is particularly important to understand the potential pathogenetic mechanisms, to find biomarkers that could be used to predict the progression of T1D and monitor disease activity, and to improve the effectiveness of therapies.

Along with most other autoimmune disorders, multiple genetic susceptibility loci contribute to T1D susceptibility [38]. In addition, nongenetic or environmental factors may contribute to the risk of T1D, given that the concordance rate for Type 1 diabetes in identical twins is not 100% [39]. T1D, therefore, has been suggested to result from a complex interplay between varying degrees of genetic susceptibility and environmental factors [40].

T1D is characterized by progressive lymphocytic infiltration of the pancreatic islets by cells of the immune system—with central roles of CD4^+^ and CD8^+^ T cells as well as macrophages [41]. This lymphocyte infiltration can result in inflammatory infiltrates within islets (insulitis) and destruction of insulin production. In some individuals, this infiltration may be held in check through regulation without overt clinical manifestations. In other cases, it can progress to a destructive immune response where *β*-cells are selectively killed. In most T1D patients, their *β*-cell mass is reduced by 70–80% at the time of diagnosis [41].

A variety of mechanisms have been proposed to contribute to *β*-cell death. *β*-Cell death is probably caused by direct contact with activated macrophages and T cells and/or exposure to soluble mediators secreted by these cells, including cytokines, nitric oxide (NO), and reactive oxygen species (ROS) [41]. Recent experimental and clinical evidence suggest that inflammatory mediators, such as cytokines, might play an important role in the pathogenesis of T1D in addition to metabolic and hemodynamic changes [42–44]. Cytokines are small-secreted proteins that facilitate the interactions and communication between cells, stimulate the proliferation of antigen-specific effector cells, and mediate the local and systemic inflammation via autocrine, paracrine, or endocrine mechanisms [45]. In this review, we discuss recent research progress in the understanding of the roles of inflammatory mediators (especially the cytokines) in the pathogenesis of T1D, which will provide insights into the molecular basis for the early defects and to further develop targeted therapies to better treat T1D.

## 4. Pathogenesis of T1D

T1D pathogenesis has been extensively studied using experimental animal models, which have greatly enhanced our understanding of the possible pathogenic features of the disease. The most commonly used models are the nonobese diabetic (NOD) mouse and the diabetes-prone BioBreeding (DP-BB) rats [46]. Although the precise mechanisms responsible for the initiation and progression of *β*-cell destruction have not been fully elucidated, it is generally believed that *β*-cell autoantigens, macrophages, dendritic cells (DC), B lymphocytes, and T lymphocytes are involved in triggering *β*-cell-specific autoimmunity [47]. The autoimmune reaction towards the *β*-cell appears to begin in the pancreatic lymph nodes (PLN), the site of islet cell-specific self-antigen presentation [48]. In PLN, T cells that have escaped negative selection in the thymus first meet *β*-cell antigens presented by dendritic cells. T cells migrate to the islets via the circulation and establish insulitis initially around the islets. Regulatory T cells of different cell surface phenotypes and cytokine secretion profiles may also be involved in modulating this unstable equilibrium [49]. Eventually, the chronic process ends in favor of the *β*-cell-reactive T cells, which eventually causes sufficient reduction of the *β*-cell mass to render the patient insulin-dependent [50].

Other than the T-cell-mediated *β*-cell destruction, there are other ways in which *β*-cell death might occur. It has been reported that there is increased *β*-cell sensitivity to cytokine-mediated killing since *β*-cells are particularly sensitive to the cytokine interleukin-1*β* (IL-1*β*) [51]. Endoplasmic reticulum (ER) stress is also a factor that increases the sensitivity of islet *β*-cells to self-directed cellular destruction [52].

## 5. Cytokines in T1D

The mechanisms involved in *β*-cell death in T1D may differ. However, many cells that have been shown to produce cytokines have been proven to participate in *β*-cell destruction in this autoimmune disease. Insulitis is an inflammatory reaction that leads to the loss of most *β*-cells after long periods of disease [53]. Several cytokines have been shown to play important roles in developing T1D at the level of both immune responses and targeting *β*-cells. TNF-*α*, IL-1*β*, and IFN-*γ* are the most likely cytokines acting in synergy during inflammation of pancreatic *β*-cells, leading to the activation of a final common pathway, such as nuclear factor-*κ*B (NF-*κ*B) and, ultimately, to *β*-cell destruction. NF-*κ*B can be activated by a variety of stimuli, including TNF-*α*, IL-1, receptor for advanced glycation end products (RAGE), and Toll-like receptors (TLRs).

## 6. Inflammatory Cytokines in T1D

### 6.1. TNF-*α*

TNF-*α* is a potent pleiotropic proinflammatory cytokine secreted by innate immune cells such as macrophages and monocytes and by differentiated T cells [54]. The evidence suggests that TNF-*α* exerts its proinflammatory effects by increasing the production of IL-1*β* and IL-6, expression of adhesion molecules, proliferation of fibroblasts and procoagulant factors, as well as initiation of cytotoxic, apoptotic, acute-phase responses, and inhibition of apoptosis [55]. TNF-*α* plays multiple roles in the development and function of the immune system and manipulation of TNF-*α* and its receptors and has revealed numerous aspects of their functions in autoimmune disease, such as T1D.

Early studies reported that, *in vitro*, a combination of TNF-*α* with IFN-*γ* resulted in the death of *β*-cells via the regulation of intraislet IL-1, NO production, and caspase activation [56, 57]. Targeted overexpression of TNF-*α* in the pancreatic islets of transgenic NOD mice accelerated the progression of diabetes [58]. Studies conducted in adoptive transfer models have suggested that TNF-*α* plays a critical role in Th1 and Th2 cells in diabetes induction [59, 60]. In accordance with these findings, anti-TNF-*α* mAb administration to newly onset T1D NOD mice prevented disease progression by restoring euglycemia, self-tolerance, and normal insulin signaling [44, 61]. However, it has been indicated that TNF-*α* might play a dual role in T1D. Administration of anti-TNF-*α* treatment at 4 weeks of age or later contributed to the accelerated progression of T1D, while the systemic administration of TNF-*α* protected against diabetes [62]. Therefore, in T1D experimental models, TNF-*α* can serve as a double-edged sword by either promoting or inhibiting inflammatory responses. The involved complex factors may include the stage of disease, the length and timing of TNF expression, and the background genetic susceptibility.

### 6.2. IFN-*γ*

IFN-*γ* is a proinflammatory cytokine that is produced in T cells and natural killer cells [63]. It has been reported that IFN-*γ* plays a key role in driving the autoimmune pathogenesis of T1D. IFN-*γ* signal transduction requires activation of the tyrosine kinases JAK1 and JAK2 that lead to the phosphorylation of STAT1, which then dimerizes and translocates to the nucleus, binding the *γ*-activated sites of diverse genes [64]. However, the precise mechanisms by which it contributes to *β*-cell autoimmunity and T1D progression are not clear yet.

Experimental studies, which were conducted in NOD mice, have reported that the blockade of IFN-*γ* function via either specific Abs or IFN-*γ* receptors prevented T1D progression and reduced its incidence [65, 66]. A recent study reported that the T1D candidate gene protein tyrosine phosphatase, type 2 (PTPN2), modulated IFN-*γ* signal transduction at the *β*-cell level and might therefore contribute to the pathogenesis of T1D [67]. Although IFN-*γ* has been generally believed to be important in autoimmune T1D pathogenesis, there are still questions regarding the role of IFN-*γ* in T1D, since the development of spontaneous *β*-cell autoimmunity does not change in NOD mice lacking IFN-*γ* or IFN-*γ* receptor expression. By employing an adoptive transfer model, it was shown that IFN-*γ* played an important role in CD4^+^ T cell-mediated destruction of *β*-cells instead of CD8^+^ T cells [68].

Despite a substantial body of evidence, there are still conflicting results in the literature about the possible role of IFN-*γ* in T1D. It has been suggested that IFN-*γ* signaling is dispensable for the development of T1D in NOD mice, and in addition, IFN-*γ* gene disruption may delay but not prevent the onset of T1D [69]. Therefore, such findings need to be repeated and confirmed by other research groups before reaching a consensus.

### 6.3. IL-1*β*

IL-1, a prototypical proinflammatory cytokine, has long been believed to cause *β*-cell dysfunction and death. There is IL-1 expression early in the insulitis infiltrate, and it may be considered as a circulating biomarker of T1D risk. The principal components of the IL-1 family are IL-1*α* and IL-1*β* [70]. IL-1*α* is located within the plasma membrane, whereas IL-1*β* is exported out of the cell [70]. IL-1*β* is an inflammatory cytokine that is produced mainly by blood monocytes and also by macrophages, dendritic cells, and a variety of other cells in the body [71].

Studies have indicated that IL-1*β* plays a major role in mediating both impaired function and destruction of pancreatic *β*-cells during the development of autoimmune T1D [72]. Secretion of insulin was inhibited by the treatment of rodent islets with IL-1*β*, followed by *β*-cell destruction [73]. In human islets, the results showed that exposure of *β*-cells to IL-1*β* or IL-1*β* plus IFN-*γ* led to *β*-cell functional changes similar to those that were observed in prediabetic patients [74]. IL-1*β* has a preferential inhibitory effect on the first phase of glucose-induced insulin release via reducing the docking and fusion of insulin granules to the *β*-cell membrane [75]. Based on pharmacological studies, p38 MAPK and c-Jun N-terminal kinase (JNK), which are members of the mitogen-activated protein kinases (MAPKs), are specifically activated in IL-1*β*–mediated *β*-cell dysfunction [76]. However, there was moderate or no protection from anti-IL-1 strategies or genetic ablation of IL-1 or IL-1RTI in animal models of T1D [77]. In contrast to this disappointing result, in a recent clinical study, it was reported that IL-1*β* pathway blockade in T1D causes a reduction in monocyte trafficking [78]. There are a few factors that may cause negative outcomes beyond the obvious possibilities, including the intervention time, the administration dosage, the C-peptide decline, and the efficacy of IL-1 antagonism [79].

## 7. Intracellular Signaling Pathways Activated by Proinflammatory Cytokines in T1D

Pancreatic *β*-cells are the targets of an autoimmune assault in T1D, with invasion of the islets by mononuclear cells in insulitis, causing loss of most *β*-cells after prolonged periods of disease [80]. During the course of insulitis, *β*-cell death results from direct contact with activated macrophages and T-cells or their secreted soluble mediators. As the main cause of *β*-cell death at the onset of T1D, apoptosis is a process that is highly regulated, activated, and/or modified by extracellular signals, intracellular ATP levels, phosphorylation cascades, and expression of pro- and antiapoptotic genes. There are transcriptional factors and signaling pathways involved in the progression of *β*-cell death. Proinflammatory cytokines play a key role in the cytokine-promoted *β*-cell “decision” to undergo apoptosis [41]. It has been identified in microarray experiments that approximately 700 genes and expressed sequence tags are up- or downregulated in purified rat *β*-cells or insulin-producing cells after exposure to IL-1*β* and/or IFN-*γ* [81, 82]. The transcriptional factors NF-*κ*B and STAT-1 are the main regulators of the pathways triggered by IL-1*β*, IFN-*γ*, and TNF-*α*.

### 7.1. IL-1*β* Signaling

The transcription factor nuclear factor kappa B (NF-*κ*B) is known to be the main mediator of IL-1*β* signaling [83]. Similar to the identified pathway by which IL-1*β* activates NF-*κ*B in various cell types and experimental models, the signaling pathway in *β*-cells involves TRNF6, JNK, and IKKs. After being produced and released by macrophages and T cells, IL-1*β* can bind to its receptor 1 (IL-1R1) on the cell surface of target cells. And then IL-1R1 can recruit IL-1 receptor accessory protein (IL-1RAcP) [84], which allows for combination of the adaptor protein myeloid differentiation factor 88 (MyD88) and the recruitment of IL-1R1-activated kinase 1 (IRAK1) and/or IRAK2 [85]. IRAK proteins are found in a complex with a protein named Tollip prior to recruitment to the receptor. Tollip transiently associates with IL-1RacP during recruitment of the Tollip–IRAK complexes to the activated receptor complex [86]. Then, the recruitment of TNF-receptor-associated factor-6 (TRAF6) to IRAK1 and IRAK2 exerts an activation of inhibitors of NF-*κ*B (I*κ*B) kinase (IKK) via NF-*κ*B-inducing kinase (NIK) [87]. IKK then phosphorylates I*κ*B and triggers the degradation and release of NF-*κ*B from the inhibitory interaction. Additionally, in response to IL-1, there is an activation of phosphatidyl inositol-3 kinase (PI3K) [88]. PI3K activity is required (but not sufficient) for NF-*κ*B activation [88].

There are several target genes that can be regulated by NF-*κ*B, including cytokines, chemokines, cell adhesion molecules, apoptosis regulators, and other transcriptional factors. NF-*κ*B signaling is highly cell type-specific. In *β*-cells, NF-*κ*B activation exerts a proapoptotic effect, while NF-*κ*B activation promotes cell survival in most cells [89]. Studies suggest that inhibition of NF-*κ*B exerts a protective effect on *β*-cells against damage *in vivo* and *in vitro* [89]. In primary rat *β*-cells treated with cytokines, there are a large number of NF-*κ*B-targeted genes that have been identified via DNA microarray technology [82]. This study also reported various genes that are regulated by cytokines after induced NF-*κ*B activation. There is upregulation of genes that are involved in immune responses (e.g., MHC-II-associated invariant chain *γ* and MHC-I) and stress responses (including CHOP, C/EBP*β* and *δ*, Hsp27, and MnSOD), while there is downregulation of genes that are involved in *β*-cell function (glucose transporter-2 (Glut-2)), insulin production (Isl-1), insulin processing (PC-1), insulin release (PLD-1, CCKA-receptor), and Ca^2+^ homeostasis (SERCA2, IP 3-kinase) [82]. It is known that iNOS is strongly induced by NF-*κ*B in both rat *β*-cells and human pancreatic islets [81, 90], which may induce the production of ROS and oxidative stress damage. In addition to NF-*κ*B, IL-1*β* signaling is also able to activate mitogen-activated protein kinase (MAPK) extracellular signal-regulated kinase (ERK) 1/2 and suppress cytokine signaling-3 (SOCS-3) [91]. MAPKs and SOCS-3-induced signaling pathways are interlinked with NF-*κ*B-regulated pathways. MAPK has been shown to potentiate IL-1*β*-dependent NF-*κ*B activation and iNOS production, while ERK 1/2 activation contributes to cytokine-induced apoptosis in rat pancreatic *β*-cells [92]. However, SOCS is believed to provide a negative feedback to affect NF-*κ*B signaling and *β*-cell death. SOCS-3 demonstrates an inhibition of IL-1*β* signaling upstream and thus downregulates all effects of IL-1*β*. There are a variety of IL-1*β*-induced proapoptotic genes suppressed by SOCS-3, most of which are NF-*κ*B-dependent [91]. SOCS was also shown to protect rat *β*-cells against IL-1*β*- and TNF*α*-induced cell death [92]. Although there have been advances in identifying NF-*κ*B-regulated genes in *β*-cells, to which extent the expression is regulated by IL-1*β* alone has not been well studied. The determination of individual targets of the cytokines will be helpful in better understanding of the role of cytokines in T1D in the future.

### 7.2. TNF-*α* Signaling

It has been demonstrated that TNF-*α* contributes to the activation of NF-*κ*B in pancreatic *β*-cells [93]. TNF-*α* binds to and activates the TNF receptor (TNFR1), which is present on the surface of *β*-cells [94]. Binding of the ligand TNF-*α* to TNFR1 leads to the activation of TNFR1, which induces the recruitment of TNF receptor-associated death domain (TRADD), TRAF2, and the death domain kinase receptor interacting protein (RIP) [95]. TRAF2 then recruits IKK to TNF-R1 while RIP mediates IKK kinase activation [96]. The activation of NF-*κ*B requires phosphorylation of I*κ*B. The activation of NF-*κ*B by TNF-*α* exerts a proapoptotic effect on rat pancreatic *β*-cells [89]. TNF-*α* signaling leads to MAPKs activation (c-Jun N-terminal kinase JNK, p38, and ERK) in a cell type-specific manner. It has been shown that in rat pancreatic *β*-cells, JNK and p38, which are induced by the TNF-*α*, exert inhibitory effects on glucose-stimulated insulin secretion and cause impaired *β*-cell function [97].

### 7.3. IFN-*γ* Signaling

Full biologic function of IFN-*γ*, the homodimeric cytokine, is mediated by the receptor complex, which consists two species-matched chains, IFN-*γ*R1 and IFN-*γ*R2 [98]. IFN-*γ*R1 is the major ligand-binding subunit, while IFN-*γ*R2 increases the IFN-*γ*R1 affinity for its ligand and plays a minor role in direct ligand binding [99]. Ligand binding leads to receptor oligomerization, with two IFN-*γ*R1 chains bound to one IFN-*γ* homodimer, and the subsequent recruitment of two IFN-*γ*R2 chains to the complex [100]. It has been known that the ligand binding results in the activation and transphosphorylation of Janus tyrosine kinase 1 and 2 (JAK1 and JAK2), which are linked with IFN*γ*R1 and IFN*γ*R2, respectively [101]. IFN-*γ* signaling is initiated by JAK2 autophosphorylation, which is followed by JAK1 phosphorylation. Then, the activated JAK1 phosphorylates IFN-*γ*R1, providing a docking site for STAT1. After binding to its receptor site, STAT1 is activated by the phosphorylation and activation of JAK2. Furthermore, STAT-1 has been shown to homodimerize and translocate to the nucleus where it stimulates target gene expression [102]. In a STAT-1^−/−^ NOD mouse model, islets treated with IFN-*γ* and TNF-*α* or IFN-*γ* and IL-1*β* were shown to be resistant to apoptosis [103]. Consistent with the results above, STAT1 blockade prevented diabetes progression in the mouse model induced by injection of multiple low doses of streptozotocin [104]. In a recent gene expression analysis, STAT-1 was found to regulate 2000 genes in response to cytokine (IL-1*β* and IFN-*γ*) exposure in *β*-cells [105]. STAT-1 is a regulator of IL-1*β*/IFN-*γ*-mediated induction of chemokines (e.g., CXCL9, CXCL10, CXCL11, and CCL20) [105]. It has been shown that *in vitro* and *in vivo* there is a reduced production of CXCL10 in response to cytokine exposure [106]. Additionally, STAT-1 is able to downregulate several genes that are specific to the functions of *β*-cells, such as glucokinase, insulin, Glut2, and prohormone convertases, as well as transcriptional factors involved in the differentiation and maintenance of the *β*-cell phenotype (e.g., Pdx1, MafA, and Nkx2.2) [107]. Finally, STAT-1 plays a key role in the regulation of genes that mediate intracellular stress and apoptotic pathways. The apoptosis-related genes that are regulated by STAT-1 include Puma, CHOP, Bid, caspase-3, -4, -7, DP5/Hrk, and endoplasmic reticulum stress-transducing genes (XBP1, ATF4) [108]. IFN-*γ* also plays an important role in inducing IL-1*β*-mediated iNOS and causing oxidative stress damage. It has been indicated that treatment of a rat insulinoma cell line (RIN-r) with a combination of IL-1*β* and IFN-*γ* could induce the mitochondrial apoptotic pathway via an iNOS-dependent manner [109]. In T1D, IFN-*γ* exerts inflammatory effects via negative feedback regulation of interferon-regulated factor-1 (IRF-1) as well as SOCS-1 and SOCS-3 [109, 110]. Via the STAT-1 regulatory role, IRF-1 exerts an inhibitory effect on chemokine expression in *β*-cells and leads to T cell infiltration of Langerhans islets [111]. Transgenic expression of SOCS-1 in *β*-cells was shown to protect *β*-cells against infiltrating autoreactive T cells and to be able to prevent diabetes development in NOD mice [112, 113]. Taken together, in *β*-cells, IFN-*γ* activates the STAT-1 signaling pathway and regulates the key processes, which are essential steps in the loss of *β*-cell function, stress, and finally death. In addition, IFN-*γ* leads to an increased sensitivity of *β*-cells to apoptotic stimuli and intracellular stress via the regulation of a number of genes.

## 8. Conclusion

During the last decade, a great deal has been learned about the role of cytokines in the autoimmune disease of T1D and depression. In this review, we have summarized the latest evidence regarding the role of cytokines and the signaling pathways involved in T1D (Figure 1) and depression. We outlined the most relevant cytokines in these two diseases to generate an idea that cytokines may be the common pathway shared by these two disorders. In addition, several signaling pathways also regulate these diseases' development through mechanisms that have not been fully investigated yet. Currently, there is uncertainty around this topic, as there have been paradoxical results regarding these cytokines while using similar or even the same models. This suggests that there is a lot more to be learned. The discovery of new cytokines and the regulatory mechanisms underlying these two diseases will help to improve our understanding in this field. Successful translation of findings from animal models to clinical trials, while adopting a mechanistic perspective, may yield new approaches for the management of T1D and depression, reducing their incidence and prevalence, limit pharmacological toxicity, and avoid global immunosuppression.

## Figures and Tables

**Figure 1:**